# tVNS Increases Liking of Orally Sampled Low-Fat Foods: A Pilot Study

**DOI:** 10.3389/fnhum.2020.600995

**Published:** 2020-11-27

**Authors:** Lina Öztürk, Pia Elisa Büning, Eleni Frangos, Guillaume de Lartigue, Maria G. Veldhuizen

**Affiliations:** ^1^Department of Anatomy, Faculty of Medicine, Mersin University, Mersin, Turkey; ^2^The John B. Pierce Laboratory, New Haven, CT, United States; ^3^Department of Psychiatry, Yale School of Medicine, New Haven, CT, United States; ^4^Department of Psychology, University of Cologne, Cologne, Germany; ^5^National Center for Complementary and Integrative Health, National Institutes of Health, Bethesda, MD, United States; ^6^Department of Pharmacodynamics, College of Pharmacy, University of Florida, Gainesville, FL, United States; ^7^Center for Integrative Cardiovascular and Metabolic Disease, University of Florida, Gainesville, FL, United States; ^8^Department of Molecular and Cellular Physiology, Yale School of Medicine, New Haven, CT, United States

**Keywords:** vagus nerve (VN) stimulation, food reward, food preferences, obesity, healthy food choice

## Abstract

Recently a role for the vagus nerve in conditioning food preferences was established in rodents. In a prospective controlled clinical trial in humans, invasive vagus nerve stimulation shifted food choice toward lower fat content. Here we explored whether hedonic aspects of an orally sampled food stimulus can be modulated by non-invasive transcutaneous vagus nerve stimulation (tVNS) in humans. In healthy participants (*n* = 10, five women, 20–32 years old, no obesity) we tested liking and wanting ratings of food samples with varying fat or sugar content with or without tVNS in a sham-controlled within-participants design. To determine effects of tVNS on food intake, we also measured voluntary consumption of milkshake. Spontaneous eye blink rate was measured as a proxy for dopamine tone. Liking of low-fat, but not high-fat puddings, was higher for tVNS relative to sham stimulation. Other outcomes showed no differences. These findings support a role for the vagus nerve promoting post-ingestive reward signals. Our results suggest that tVNS may be used to increase liking of low-calorie foods, which may support healthier food choices.

## Introduction

The vagus nerve (VN) carries signals about food from gut to brain (Berthoud, 2008; Lartigue, 2016; Yuan and Silberstein, 2016a). The role of the VN in food consumption is classically thought to mostly entail establishing satiety by transducing signals from mechanoreceptors in the stomach and the release of anorexigenic hormones into the blood (Berthoud, 2008). Recently several pre-clinical studies have dissected a role for vagus nerve signals beyond satiety. Williams et al. (2016) show that vagal sensory neurons are capable of sensing a range of metabolic stimuli from the gut, including macronutrients (Williams et al., 2016). Opto- and chemogenetic experiments showed that nodose vagal sensory neurons are necessary for post-ingestive fat mediated reward (Han et al., 2018). A population of neurons in brainstem that receive sugar signals from the vagus were chemogenetically activated to create preferences to otherwise less-preferred sweet stimuli (Tan et al., 2020). Together, these pre-clinical findings reveal a gut-to-brain post-ingestive fat and sugar-sensing pathway critical for the development of food preference. If these data translate to humans, these findings would support targeting the VN to modulate food preferences.

Vagal nerve stimulation (VNS) involves implanting electrodes on the vagus nerve and using electrical pulses to generate firing potentials (Yuan and Silberstein, 2016b). Interestingly, implantation of a VN stimulation device for treatment of epilepsy or depression is in some retrospective studies accompanied by significant weight loss in humans (Burneo et al., 2002; Ogbonnaya and Kaliaperumal, 2013), and in a prospective study decreased preference of sweet food images (Bodenlos et al., 2007). Non-invasive VNS via the auricular branch (transcutaneous VNS, tVNS) (Ellrich, 2011) is effective in treating depression in clinical studies (Kong et al., 2018), and has enabled experimental studies in healthy human participants (Frangos et al., 2015; Yakunina et al., 2017).

Recent work using tVNS modulation of responses to food stimuli in humans have shown mixed results. tVNS had no effect on electro-encephalogram responses to visual food stimuli relative to other objects, as well as no effect on food intake when tVNS was applied immediately prior to the test session (Obst et al., 2020). However, in an effort allocation task, *concurrent* tVNS increased participants' drive to obtain less-wanted prospective food rewards (Neuser et al., 2020). Two weeks of tVNS concurrent with bottle-feeding improved oral intake in about half of premature or brain injured infants who had failed oral feeding until that time (Badran et al., 2020). These results suggest that concurrent tVNS may affect hedonic responses to orally sampled foods and food intake, which has not been examined to our awareness.

Our first aim was to test the feasibility of using non-invasive VNS via the auricular branch during food consumption. Our second aim was to explore the ability of VNS to change hedonic responses to food. More specifically we asked: does tVNS (relative to sham stimulation) affect liking and wanting of orally sampled fatty and sweet foods, spontaneous eye blink rate (SEBR), and *ad libitum* milkshake consumption? These outcome variables were chosen to cover a range of assays that are thought to reflect food reward responses, including consciously experienced pleasantness (liking ratings) and motivation (wanting ratings), a physiological proxy for dopamine tone (SEBR), and a behavioral measure of motivation (*ad libitum* consumption). If VN responses to food induce dopamine mediated reward responses, we predict that tVNS relative to sham stimulation will increase hedonic responses.

## Methods

### Participants

Eleven healthy, non-smoking participants [six women, five men] with a mean (± standard deviation) age of 27.0 (± 4.0) years [range: 20–32], with a mean body mass index (BMI) of 23.2 ± 3.8 kg/m^2^ [range: 18.9–28] participated in the study. Participants were recruited through advertisements around Yale University and the city of New Haven. The Yale University School of Medicine Human Investigation Committee approved the informed consent form, which was subsequently obtained from all study participants. All participants reported having no known taste, smell, neurological, psychiatric (including eating disorders), or other pathological disorders. One of the participants was excluded because they reported not feeling any stimulation during one of the two sessions and a loose electrode was observed by the experimenter after the session. The remaining ten participants (five women and five men) were 27.5 ± 4.0 years old with a BMI of 23.1 ± 3.9 kg/m^2^.

### Design and Procedure

We used a within-participants design in which all participants were exposed to the tVNS and sham conditions on separate days, in a counterbalanced order. Participants were scheduled for the same time of day for each session. Upon arrival to the laboratory, breath alcohol levels (Alcohawk Elite Breathalyzer), urine toxicology for opiates, cocaine, THC, PCP, and barbiturates (Integrated E-Z Split Key Cup II, Innovacon Inc., San Diego, CA) and aurine pregnancy test were measured (no participants excluded). Participants were asked to arrive neither hungry nor full and were asked to rate their hunger level upon arrival using a visual analog scale (VAS; 0 = “I am not hungry at all” and 100 = “I have never been more hungry”). VAS's were also used to rate fullness and thirst. These “internal state” ratings are analyzed and reported in the Supplementary Table 1 and Supplementary Figure 1.

**Figure 1:**
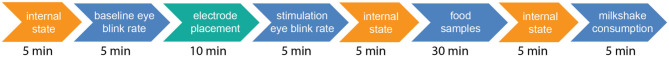
Order of tasks in a test session and approximate timing in minutes. Upon arrival at the laboratory, participants rated their hunger, fullness and thirst (“internal state”). Then a baseline spontaneous eye blink rate (SEBR) was assessed, then the stimulation device was turned on and the intensity for sham or tVNS stimulation was adjusted. Next VNS stimulation perception was assessed. Then the rest of the tasks were performed concurrent with stimulation: another SEBR measurement, internal state ratings, rating of food samples, internal state ratings and last *ad libitum* milkshake consumption. This entire procedure was repeated on two testing days, one session using sham stimulation on the earlobe, and one session using tVNS stimulation on the cymba conchae (order of sessions counterbalanced over participants).

Each session included the same events in the same order (Figure 1). First participants were outfitted with the electrodes for electro-oculogram (EOG) measurement and tVNS stimulation. Next, the participant completed a baseline spontaneous eye blink rate (SEBR) measurement. Then we adjusted tVNS stimulation intensity for each participant individually (see tVNS section below for details). Then we started a second SEBR measurement concurrent with tVNS stimulation. We then asked participants to rate their hunger, fullness and thirst a second time. Then the participants completed the fat and sweet food sampling task, and afterward rated hunger, fullness and thirst a third time. Last, they were asked to *ad libitum* consume a milkshake. All tasks were completed concurrent with tVNS stimulation. We used a single-blind design; the participant was not informed of the goal of stimulating the vagus nerve or differential innervation of the ear by the vagus nerve, while the experimenter and data-analyst were not blinded. We did not assess whether participants were aware of the association between ear location and vagus nerve innervation upon debriefing at the end of the study, thus success of the single-blind procedure was not explicitly confirmed.

#### SEBR Task

We used the EOG Pod (ADInstruments) for eye blink rate measurement. The EOG Pod utilizes the steady corneal-retinal electrical potential to detect eye movement and position. This task has been associated with DA signaling using PET (Groman et al., 2014) and with DA-dependent cognitive functions, including reinforcement learning (Jongkees and Colzato, 2016). To measure electrooculography (EOG), three Ag/AgCl self-adhesive electrodes were placed: (1) one above and another below the eye to record the vertical movements; and (2) one over a neutral point (vertebrae on the back of the neck), which is acting as a reference electrode. Participants were asked to look at a printed black fixation cross on a white poster board at 75 cm distance in an artificially lit room (blinds closed) with controlled humidity (50%) and room temperature (23°C). Participants were not instructed in any manner about blinking. Eye blink rate was taken as the mean number of blinks per minute during a 5 min measurement period.

#### tVNS/Sham Stimulation

Mild transcutaneous electrical stimulation was applied counterbalanced to either the cymba conchae of the left ear (tVNS) or the left ear lobe (sham) on separate visits using a commercially available TENS unit (transcutaneous electrical nerve stimulator, Twin Stim® Plus 3rd edition, Roscoe Medical Inc.) attached to a pair of silver electrodes. The electrodes are mounted on a round plastic stabilizer that fits into the cavum with adjustable distance between stabilizer and electrodes (similar to the Cerbomed Nemos® device). For the tVNS condition, the electrodes are positioned into the cymba conchae with electrode gel on each of the electrodes and a piece of medical tape was used to secure the electrode mount. For the sham condition, the entire earpiece was turned 180° to place the electrode on the ear lobe, and again taped into place. Both sham and tVNS stimulation used the following parameters: a biphasic square wave pulse at 25 Hz and a pulse width of 250 μs, with a duty cycle of 30 s on, 30 s off. The total stimulation duration was ~45 min, the time needed to complete the tasks from second SEBR measurement through *ad libitum* consumption. Stimulation was applied with constant voltage. These parameters are reported in agreement with proposed reporting guidelines (Farmer et al., 2020). The amplitude of stimulation was calibrated for each session (stimulation location) and participant individually with a procedure commonly used (Kaniusas et al., 2019; Farmer et al., 2020), intended to adjust the amplitude to the highest stimulation level that can be reached without causing pain or discomfort. Since there are tissue and innervation differences in the sham and tVNS locations, the resulting stimulation amplitudes may differ between sites, but the calibration ensures that the sensation between sites remains comparable, thus controlling for perception-related placebo effects. While the stimulation was gradually increased, the participant was asked to report when a “pricking, stinging or burning” sensation was felt, which indicated their pain threshold. The stimulus intensity was then immediately decreased gradually until the participant reported an innocuous, comfortable “tingling, vibrating or drumming” sensation. The intensity of the stimulus remained at that selected level for the duration of the session unless the participant reported discomfort, in which case, the stimulus intensity was decreased in the same manner as during calibration to relieve discomfort. For one participant we reduced the intensity of the sham stimulation, which was requested after tasting the puddings and before tasting the Jell-O's. To confirm iso-intense stimulation, participants rated the intensity of the sensation on a General Labeled Magnitude Scale (described in detail in the next section). This type of stimulation is safe and well-tolerated (Redgrave et al., 2018).

#### Fat and Sweet Food Samples Task

Participant were asked to sample and rate flavor stimuli with varying fat content (puddings) and varying sugar content (Jell-O's). All stimuli were made from commercially available ingredients. Pudding samples were prepared with 0, 3.1, 6.9, and 15.6% fat weight by weight (w/w). The samples were prepared by mixing instant pudding (vanilla or chocolate flavored, Kraft Foods) in varying proportions of milk and heavy cream (Guida's Dairy, Connecticut) to varying fat content. The sugar content was held constant between the four stimuli at 4.6% (w/w). Jell-O samples were prepared by mixing unflavored gelatin powder (Jell-O, Kraft Foods) with Kool-Aid orange or strawberry unsweetened powdered flavor with 0, 0.1, 0.56, and 1 molar (M) sucrose (Sigma-Aldrich) concentration solutions. Each participant was asked which flavors they preferred to receive during scheduling of their appointment. Each stimulus was presented three times. First the puddings were presented in a randomized order within a block of 12 stimuli. Then the Jell-O samples were presented in a randomized order in a second block of 12 stimuli. A trial started with the experimenter cueing the participant to close their eyes (to eliminate color cues signaling content). The participant held out their hand and the experimenter placed a small ice-cream sampling spoon with a volume of about half a teaspoon of the food sample on it. The participant then placed the entire sample in their mouth and swallowed the sample. Then they opened their eyes and made ratings of the following attributes in this order: overall intensity, (dis)liking, sweetness, saltiness, fattiness, creaminess, oiliness and wanting. Each participant had previously been trained to use the General Labeled Magnitude Scale (gLMS) to rate overall intensity, saltiness and sweetness, the Labeled Hedonic Scale (LHS) to rate liking or disliking, and the VAS to rate oiliness, fattiness, creaminess, and wanting of the food samples. The gLMS is a computerized psychophysical tool that requires subjects to rate the perceived intensity of a stimulus along a vertical axis lined with adjectives that are spaced quasi-logarithmically on the basis of experimentally determined intervals to yield ratio quality data (Green et al., 1996). The LHS was derived using similar methods as the gLMS but asks subjects to rate hedonic liking or disliking (Lim et al., 2009). The wanting VAS was presented with the question “how much do you want to eat this at the end of the experiment?.” The left anchor was labeled with “I would never want to eat this” and the right anchor with “I would want to eat this more than anything.” Here the hedonic (dis)liking ratings and wanting ratings were of primary interest and the other scales were included to prevent “dumping,” bias effects that occur when participants are not asked to rate important attributes that are clearly present in a stimulus. For completeness we visualize the ratings on all other scales in the Supplementary Figures 2, 3, but we include only liking and wanting ratings of the puddings and Jell-Os in the statistical analyses, given our predictions. After making these ratings, the participant rinsed their mouth with demineralized water, expectorated the water into a sink, and then the experimenter initiated a timer for a 30-s interval until the start of the next trial. This task took about 25–30 min.

#### Ad Libitum Milkshake Consumption

Finally, while the experimenter left the room to retrieve a participation-fee receipt to sign, the participant was given a carton milkshake cup (with lid and straw) with ~700 g of chocolate milkshake and the instruction “to consume as much as you want”. The experimenter returned to the testing room after 5 min. This milkshake was mixed from 1,000 ml of whole (full fat) milk (Guida's Dairy, Connecticut), 200 ml heavy cream (Guida's Dairy, Connecticut) and six tablespoons of “Chocolate Moo-usse” hot chocolate dry powder mix (Silly Cow Farms, Vermont). This mixture was stored in a refrigerator for up to 2 days and served cold (~4°C). The resulting milkshake had an approximate fat content of 8.27% (w/w) and a caloric density of ~1.2 kcal/g. The milkshake cup was weighed before and after consumption on a 1,000 g scale (Ohaus) with a precision of 1 g.

### Data Analysis

EOG signal was analyzed in LabChart8.1.13 (ADInstruments). We applied a high frequency filter and counted each excursion from a threshold determined per participant per min. Events triggered by this calculation were verified with visual inspection. The frequency of blinks per min was averaged across the duration of the 5 min of the baseline and the stimulation period. The frequency during the stimulation period was normalized per participant to their own baseline and expressed as % baseline.

Ratings for puddings and Jell-Os were averaged across three replications for each variation and then averaged across the two lower concentrations and the two higher fat and sweet concentrations.

For the *ad libitum* consumption task we subtracted the post from the pre-consumption weight and expressed as % of the pre-consumption weight.

**Figure 2:**
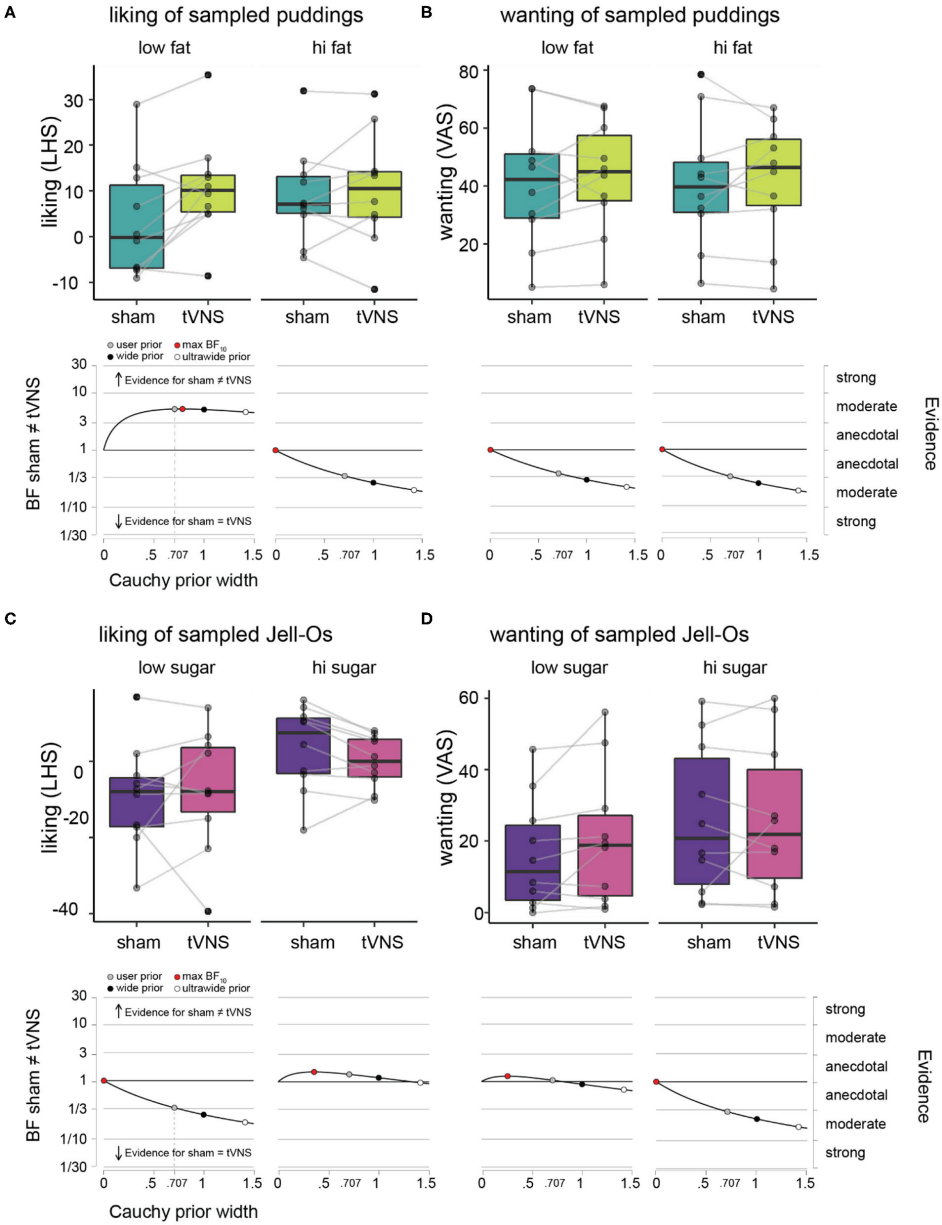
Hedonic ratings of puddings and Jell-Os during sham vs. tVNS. **(A)** Liking ratings on LHS under sham (dark green) vs. tVNS (light green), plotted for the low fat puddings (upper left panel) and high fat puddings (upper right panel) separately. The boxplots indicate central tendencies and spread of the ratings, as follows: median (middle bar in box), first and third quartiles (lower and upper hinge), 1.5 × the interquartile range (top and bottom whiskers) and outlying points (separate solid black dots outside the whiskers). We overlaid individual data points on the boxplots (transparent gray dots) and connected the dots of an individual participant between the sham and tVNS bars to make it easier to inspect the difference within a single participant. Robustness check illustrating the effects of assigning more conservative Cauchy priors (wide and ultrawide, black and white circles, respectively) relative to the default user prior (gray circle) on Bayes factor values for the effect of sham vs. tVNS for liking ratings, plotted for the low fat puddings (lower left panel) and high fat puddings (lower right panel). **(B)** Wanting ratings for puddings on VAS under sham vs. tVNS and robustness checks for effect of sham vs. tNVS on wanting ratings. Details as in **(A)**. **(C)** Liking ratings on LHS under sham (purple) vs. tVNS (pink), plotted for the low sugar Jell-Os (upper left panel) and high sugar Jell-Os (upper right panel) separately. Details as in **(A)**. **(D)** Wanting ratings for Jell-Os on VAS under sham vs. tVNS and robustness checks for effect of sham vs. tNVS on wanting ratings. Details as in **(A)**.

We then used JASP 0.12.2 to compare the effect of stimulation condition (tVNS vs. sham) on the dependent variables (stimulation amplitude, perceived stimulation intensity, liking and wanting of low and high fat/sweet food samples, SEBR, and consumption) with Bayesian paired *t*-tests (Rouder et al., 2009) and Student's paired *t*-tests. Since prior information is absent, we used the default Cauchy prior width of 0.707 (Ly et al., 2016). To examine the extent to which our conclusions depend on that prior, we report BF robustness using a wide and ultrawide prior, as well as the prior associated with the maximum BF (Carlsson et al., 2017). We tested the hypothesis that tVNS ≠ sham (H1) vs. tVNS = sham (H0) and examined Bayes Factor (BF). A BF below 1 would be interpreted as evidence in favor of H0 relative to H1, while a BF above 1 is interpreted as evidence in favor of H1 relative to H0 (Lee and Wagenmakers, 2014). Further BF interpretations are illustrated in Figure 2. and Table 1. Here we regard any relative evidence greater than “anecdotal” in favor of H1 (BF > 3) or in favor of H0 (BF < 1/3) as meaningful. These procedures follow the JASP guidelines for conducting and reporting a Bayesian analysis (van Doorn et al., 2020). For Student's paired *t*-tests, we used an alpha of 0.05. The data, JASP analysis files and results files are available online (https://osf.io/njvw5/).

**Table 1:** Descriptive statistics, Bayesian statistics, frequentist statistics.

	**sham**		**tVNS**		**Bayesian statistics**		**Frequentist statistics**	
	**ave**	**sd**	**ave**	**sd**	**BF10**	Evidence descriptor^*^	**T-statistic**	***p*-value**
**Stimulation parameters**								
Stimulation amplitude (in mA)	5.9	3.1	12.4	5.6	50.31	Very strong evidence for H1 relative to H0	−3.753	0.005
Perceived intensity of stimulation	13.3	4	13.8	5	0.32	moderate evidence for H0 relative to H1	−0.638	0.54
**Pudding ratings**								
Liking puddings low fat	3.3	12.4	10.8	11.1	5.24	moderate evidence for H1 relative to H0	3.119	0.012
Liking puddings high fat	9.1	10.4	10.3	12.3	0.36	anecdotal evidence for H0 relative to H1	0.609	0.558
Wanting puddings low fat	41.3	22.3	43.3	19.6	0.38	anecdotal evidence for H0 relative to H1	0.711	0.495
Wanting puddings high fat	40.8	22.1	42.0	20.5	0.33	borderline moderate/anecdotal evidence for H0 relative to H1	0.398	0.7
**Jell-O ratings**								
Liking Jell-O low sugar	−9.4	13.7	−7.5	15.7	0.34	anecdotal evidence for H0 relative to H1	0.499	0.63
Liking Jell-O high sugar	3.6	11.2	0.0	6.7	1.35	anecdotal evidence for H1 relative to H0	−2.043	0.071
Wanting Jell-O low sugar	16.0	15.6	20.6	19.0	1.05	borderline anecdotal evidence for H0 relative to H1/anecdotal evidence for H1 relative to H0	1.832	0.1
Wanting Jell-O high sugar	25.8	21.1	26.0	21.3	0.31	moderate evidence for H0 relative to H1	0.067	0.948
**Other measures**								
SEBR (% baseline)	12.6	48.7	35.3	50.0	0.54	anecdotal evidence for H0 relative to H1	−0.392	0.704
*Ad libitum* consumption (% total weight)	36.4	34.7	39.1	35.8	0.33	borderline moderate/anecdotal evidence for H0 relative to H1	−1.177	0.269

* *All evidence descriptors are relative, so moderate evidence in favor of H1 is relative to evidence in favor of H0. H1, tVNS ≠ sham; H0, tVNS = sham*.

## Results

### Feasibility

We visually observed head movement throughout the sessions, particularly during expectoration of water in the food sampling task, however, feasibility was confirmed by the ability to complete 21 out of 22 sessions (one session with a loose electrode) without adverse effects. As a result of a loose stimulation electrode during one session, we excluded one participant's data from our data analyses.

During sham stimulation, the stimulation amplitude was lower than during tVNS (very strong evidence in Bayesian paired *t*-test, Table 1), however, the perceived intensities were not different (moderate evidence, Table 1), as intended by the calibration procedure.

### Liking and Wanting of Pudding Samples

Average liking and wanting ratings per participant and descriptive statistics across participants for the pudding samples with varying fat content are given in Table 1 and Figures 2A,B. Under sham stimulation, participants rated low fat puddings on average between the labels “neutral” and “like slightly” (Figure 2A). The high fat puddings were rated between the labels “like slightly” and “like moderately” (Figure 2B). Under tVNS, the average liking for the low fat puddings increased on average 7.5 points relative to sham and numerically similar to the ratings for the high fat puddings under sham or tVNS stimulation. Under tVNS, the high fat puddings were still rated between the labels “like slightly” and “like moderately.” Bayesian paired *t*-test showed moderate evidence in support of liking ratings of low-fat stimuli for tVNS being dissimilar from sham stimulation relative to the hypothesis that they are similar (Figure 2A and Table 1). Anecdotal evidence was observed for high-fat stimuli being similar in liking relative to the hypothesis that they are dissimilar (Figure 2A and Table 1).

Under sham stimulation, the average wanting of low-fat puddings was numerically slightly lower than under tVNS (Figure 2B and Table 1). A similar pattern was observed for the high-fat puddings. Bayesian paired *t*-test showed anecdotal evidence was observed for high-fat stimuli being similar in wanting relative to the hypothesis that they are dissimilar (Figure 2B and Table 1).

Summarizing, tVNS increased liking ratings of low-fat stimuli by a meaningful amount from close to “neutral” to above “like slightly,” which is similar to the liking ratings that high fat puddings received, while tVNS did not affect “wanting” of puddings.

### Liking and Wanting of Jell-O Samples

Average liking and wanting ratings per participant and descriptive statistics across participants for the Jell-O samples with varying sugar content are given in Table 1 and Figures 2C,D. Under sham stimulation, low sugar Jell-O samples are rated between “slightly disliked” and “moderately disliked.” Under tVNS these ratings increased slightly numerically. Bayesian paired *t*-test showed anecdotal evidence was observed for low sugar stimuli being similar in liking relative to the hypothesis that they are dissimilar under tVNS vs. sham (Figure 2C and Table 1). The high sugar stimuli were rated slightly above “neutral” in liking under sham, and numerically decreased slightly under tVNS. Bayesian paired *t*-test showed anecdotal evidence was observed for high sugar stimuli being dissimilar in liking relative to the hypothesis that they are similar under tVNS vs. sham.

Wanting ratings for low sugar Jell-O samples numerically slightly increased under tVNS vs. sham (Figure 2D and Table 1), but with a BF of ~1 there was no evidence in favor of either hypothesis. The wanting ratings for high sugar Jell-O samples stayed numerically similar, confirmed by a Bayesian paired *t*-test that showed moderate evidence in favor of the high sugar stimuli being similar in wanting relative to the hypothesis that they are dissimilar under tVNS vs. sham.

Summarizing, tVNS did not affect liking or wanting of the low or high sugar Jell-O samples.

### Spontaneous Eye Blink Rate

**Figure 3:**
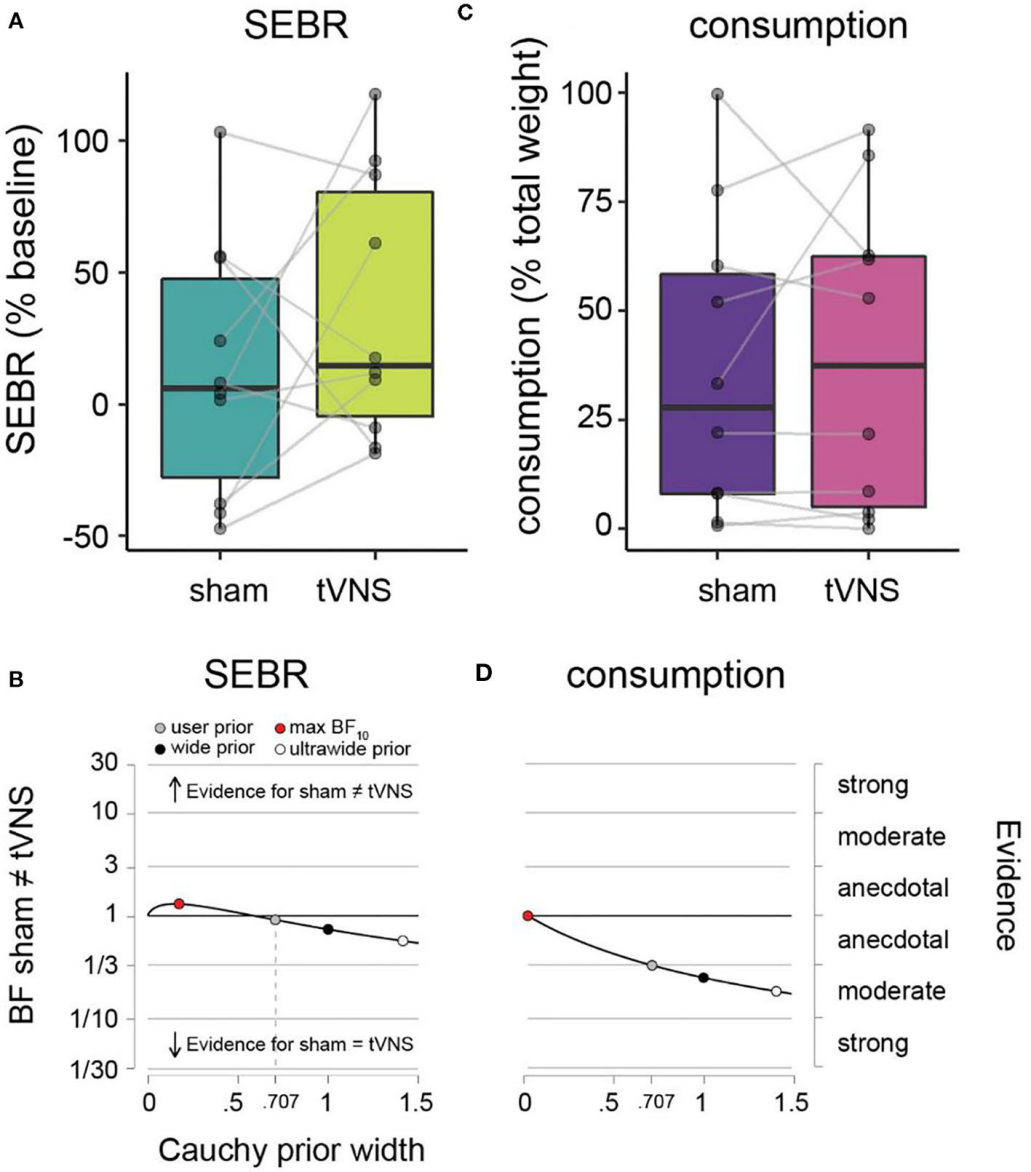
SEBR and *ad libitum* consumption during sham vs. tVNS. **(A)** SEBR (relative to baseline) under sham (dark green) vs. tVNS (light green). **(B)** Robustness checks for effect of sham vs. tNVS on SEBR. **(C)** *Ad libitum* consumption (relative to total weight) under sham (purple) vs. tVNS (pink). **(D)** Robustness checks for effect of sham vs. tVNS on consumption. Details as in Figure 2.

Average SEBR per participant and descriptive statistics across participants are given in Table 1 and Figure 3A. Under sham stimulation, participants on average had a 12.6% (± 48.7%) increase in their SEBR relative to baseline, while under tVNS the average was 35.3% (± 50.0%). No meaningful evidence for differences in favor of H0 or H1 was observed (Figure 3B and Table 1).

### *Ad libitum* Consumption

Average amount of milkshake consumption per participant and descriptive statistics across participants are given in Table 1 and Figure 3C. Under sham stimulation, participants on average consumed 36.4% (± 34.7%) of the milkshake, while under tVNS the average was 39.1% (± 35.8%). We observed meaningful evidence in favor of H0 relative to H1, such that sham and tVNS have similar effects on milkshake consumption (Figure 3D and Table 1).

## Discussion

The aim of this study was to test the feasibility of using tVNS during food consumption and to test the prediction that tVNS relative to sham stimulation will increase hedonic responses to food. In a small sample of 10 participants we observed an increased liking of low fat, but not high fat foods under tVNS vs. sham stimulation. We observed no effects on wanting of fat foods, and no effects on liking or wanting of foods varying in sugar content.

Concerning feasibility, we observed head movement during food sampling. However, in only 1 out of 22 sessions a loose electrode was observed. Various improvements may prevent the loss of contact between skin and electrode during eating movements including improved earpieces that mount behind the ear like eye glasses, tacky electrode gel, and more flexible wires.

We observed an increased liking of low fat (but not for high fat foods) under tVNS vs. sham stimulation. This is consistent with observations from pre-clinical and clinical studies with invasive VNS in humans [reviewed by Lartigue (2016)]. In humans dopamine in the dorsal striatum, measured by PET imaging of raclopride binding, positively correlates with meal pleasantness (Small et al., 2003). In animal studies, dopamine release in the dorsal striatum acts as a proxy of caloric value and drives conditioned place and flavor learning and motivated behavior (Sclafani et al., 2011; Tellez et al., 2016). In mice, stimulation of vagal sensory neurons innervating the gut drive the same reward behaviors (Han et al., 2018). Thus, one possible mechanism of action of tVNS in this study includes increased dopaminergic tone, masking the signal of phasic dopamine release to individual post-ingestive stimuli, and normalizing the comparative liking between low and high fat puddings. However, vagal afferent stimulation has been implicated in modulating a range of neurotransmitters (Hulsey et al., 2016, 2019) and affects mood, memory and cognition [reviewed by Frangos et al. (2017)]. Thus, further work is required to determine the mechanisms and downstream brain circuits that are recruited by tVNS.

We also observed that tVNS did not change liking or wanting for stimuli varying in sugar content. This is in line with the observation that vagal deafferentation of the gut results in increased food intake after a fat but not sugar preload (McDougle et al., 2020), and may suggest a more prominent role for the vagus nerve in signaling fat content to the brain. However, it is also possible that the fixed order of non-fatty sweet Jell-O samples presentation after fatty puddings samples may have worked against observing effects on sweetness perception, as a reduction in fat levels may result in a reduction of perceived sweetness (Wiet et al., 1993; Biguzzi et al., 2014). Future studies should consider using for example a factorial design with interleaved trials to manipulate sugar and fat content (Smith et al., 2020).

Dietary fat plays an important role in the development and treatment of obesity, suggesting that tVNS is an interesting avenue for modulating food preferences to shift choices toward healthier, lower fat foods in the treatment of obesity for example. However, as VNS effects may be weight dependent (Pardo et al., 2007; Obst et al., 2020), future studies should examine liking and wanting to consume food in participants with overweight and obesity.

We did not observe differences for SEBR (dopamine tone proxy) or *ad libitum* consumption (satiety and/or motivated behavior). Our specific choice of behavioral assays could not confirm dopamine release as the mechanism of the low-fat food preference shift. However, this does not exclude dopamine release or motivated behavior as a mechanism. For example, recently tVNS was shown to modulate motivated behavior by increasing participants' drive to approach less-wanted rewards (Neuser et al., 2020) and SEBR may not be a good proxy for dopamine function in humans (Dang et al., 2017). We also cannot rule out that the population of neurons that innervates the ear rather than the gut is not the right vagal population to stimulate nor that the choice of ABVNS location in the cymba conchae or stimulation parameters are suboptimal (Badran et al., 2018a,b). Our study has various other limitations, most importantly the small sample size. Independent studies and larger sample sizes will be necessary. Future studies should also assess success of the blinding procedure and prior knowledge of vagal innervation of the ear by the participant, as recently recommended (Farmer et al., 2020).

In conclusion, we observed preliminary evidence in support of tVNS' capability to modulate liking of low fat foods, which may support behavioral choices for healthier foods.

## Data Availability Statement

The datasets presented in this study can be found in online repositories. The names of the repository/repositories and accession number(s) can be found here: https://osf.io/njvw5/.

## Ethics Statement

The studies involving human participants were reviewed and approved by Yale University School of Medicine Human Investigation Committee. The patients/participants provided their written informed consent to participate in this study.

## Author Contributions

MV and GdL conceived the study. MV, EF, and GdL designed the study. MV and PB collected the data. MV performed the statistical analyses. LÖ wrote the first draft of the manuscript. All authors contributed to manuscript revision, read, and approved the submitted version.

## Conflict of Interest

The authors declare that the research was conducted in the absence of any commercial or financial relationships that could be construed as a potential conflict of interest.
