# Supplementary material for: tVNS Increases Liking of Orally Sampled Low-Fat Foods: A Pilot Study
Source: Front Hum Neurosci. 2020 Nov 27;14:600995. doi: 10.3389/fnhum.2020.600995 (PMC7731579; doi:10.3389/fnhum.2020.600995)
Supplement: Supplementary file 1 [file Data_Sheet_1.PDF]

SupplementaryTable 1.

Descriptive statistics, Bayesian statistics, frequentist statistics for internal state ratings (hunger, fullness, thirst)

	**sham**		**tVNS**		**Bayesian statistics**		**Frequentist statistics**	
	**ave**	**sd**	**ave**	**sd**	**BF10**	**Evidence descriptor***	**T-statistic**	**p-value**
Internal state ratings at arrival								
**Hunger§**	34.67	14.75	39.51	13.70	0.807	anecdotal evidence for H0 relative to H1	-1.551	0.165
**Fullness§**	54.70	19.18	50.12	18.45	0.367	anecdotal evidence for H0 relative to H1	0.456	0.662
**Thirst§**	55.18	17.26	59.61	5.83	0.419	anecdotal evidence for H0 relative to H1	-0.734	0.487
Internal state ratings before food samples								
**Hunger**	47.77	19.55	50.15	13.93	0.341	anecdotal evidence for H0 relative to H1	-0.48	0.643
**Fullness**	37.69	19.84	44.37	16.18	0.561	anecdotal evidence for H0 relative to H1	-1.223	0.252
**Thirst**	59.02	16.93	55.76	9.44	0.346	anecdotal evidence for H0 relative to H1	0.519	0.616

Internal state ratings after food samples								
**Hunger**	23.24	13.74	21.25	26.14	0.315	moderate evidence for H0 relative to H1	0.212	0.837
**Fullness**	12.96	5.35	22.88	17.72	1.075	borderline anecdotal evidence for H0 relative to H1/anecdotal evidence for H1 relative to H0	-1.849	0.097
**Thirst**	15.96	10.05	26.46	25.26	0.506	anecdotal evidence for H0 relative to H1	-1.104	0.298

* All evidence descriptors are relative, so moderate evidence in favor of H1 is relative to evidence in favor of H0. H1: tVNS ≠ sham, H0: tVNS = sham

§ Data missing for two participants.

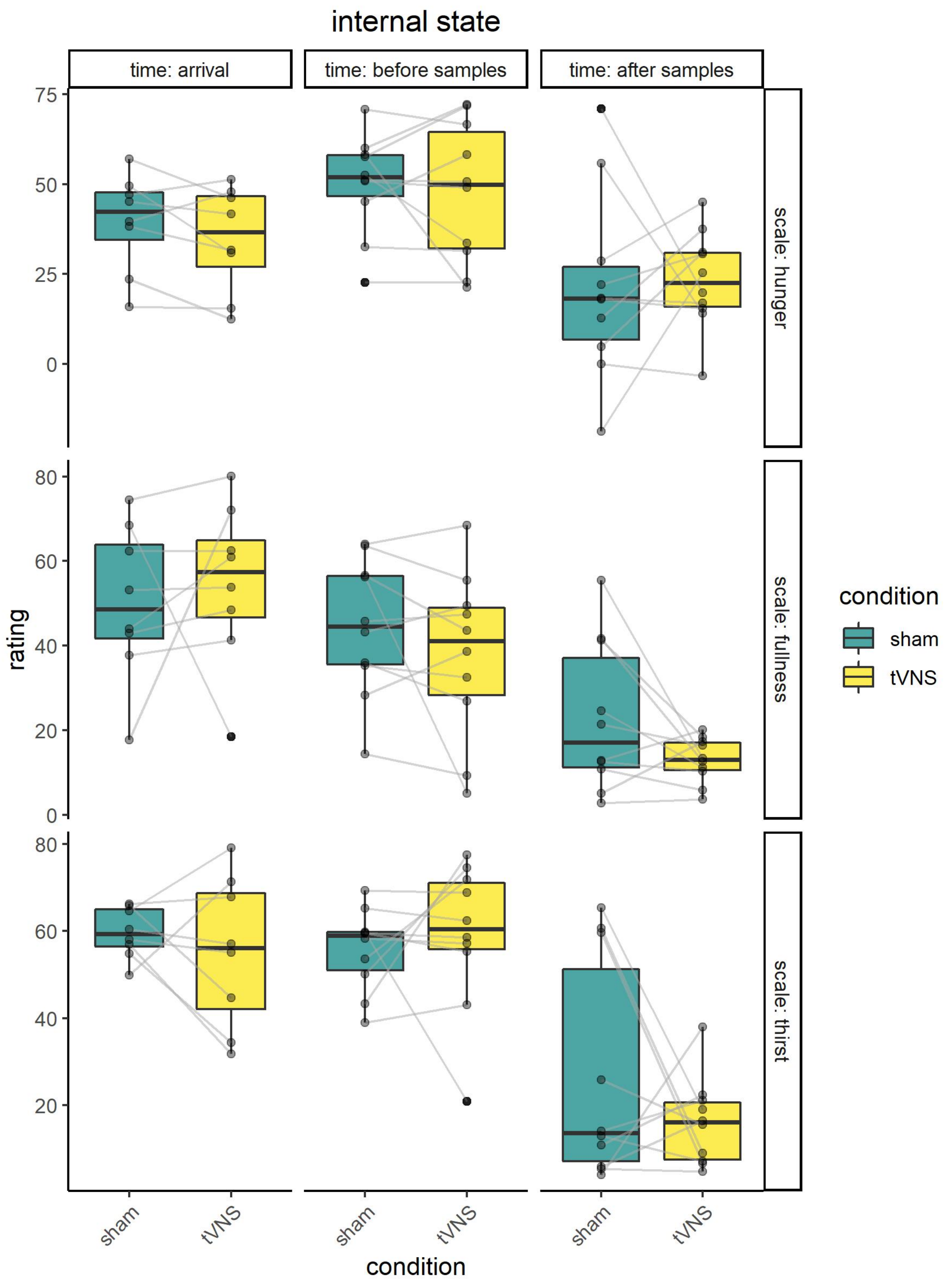

Supplementary Figure 1: Internal state ratings under sham (dark green) vs tVNS (light green), plotted for the hunger ratings (top row), fullness ratings (middle row) and thirst ratings (bottom row), and three timepoints, upon arrival at the laboratory (left column), before tasting the food samples (center column) and after tasting the food samples (right column) separately. The boxplots indicate central tendencies and spread of the ratings on a visual analog scale, as follows: median (middle bar in box), first and third quartiles (lower and upper hinge), 1.5 x the interquartile range (top and bottom whiskers) and outlying points (separate solid black dots outside the whiskers). We overlaid individual

data points on the boxplots (transparent grey dots) and connected the dots of an individual participant between the sham and tVNS bars to make it easier to inspect the difference within a single participant

Supplementary analyses for internal state ratings:

We used JASP 0.12.2 to compare the effect of stimulation condition (tVNS vs sham) on the dependent variables (hunger, fullness, thirst) with Bayesian paired t-tests (Rouder et al., 2009) and Student's paired t-tests. Since prior information is absent, we used the default Cauchy prior width of .707 (Ly et al., 2016) for the Bayesian statistical tests. To examine the extent to which our conclusions depend on that prior, we report BF robustness using a wide and ultrawide prior, as well as the prior associated with the maximum BF (Carlsson et al., 2017). We tested the hypothesis that tVNS ≠ sham (H1) vs tVNS = sham (H0) and examined Bayes Factor (BF). A BF below 1 would be interpreted as evidence in favor of H0 relative to H1, while a BF above 1 is interpreted as evidence in favor of H1 relative to H0 (Lee & Wagenmakers, 2014). Further BF interpretations are illustrated in Fig 2. and Table 1. Here we regard any relative evidence greater than "anecdotal" in favor of H1 (BF > 3) or in favor of H0 (BF < 1/3) as meaningful. These procedures follow the JASP guidelines for conducting and reporting a Bayesian analysis (van Doorn et al., 2019). For Student's paired t-tests, we used an alpha of .05. The data, JASP analysis files and results files are available online (https://osf.io/njvw5/).

For two participants, due to an oversight, internal state ratings were not collected at the time of arrival at the laboratory. Bayesian paired t-tests showed anecdotal evidence for hunger, fullness and thirst being similar under tVNS and sham relative to the hypothesis that they are dissimilar (Supplementary Table 1) at all timepoints, with the exception of only post-sample fullness ratings showing Bayes factor in the range of "anecdotal evidence" or what may be regarded a trend (p-value <.1) for a difference between sham and tVNS .

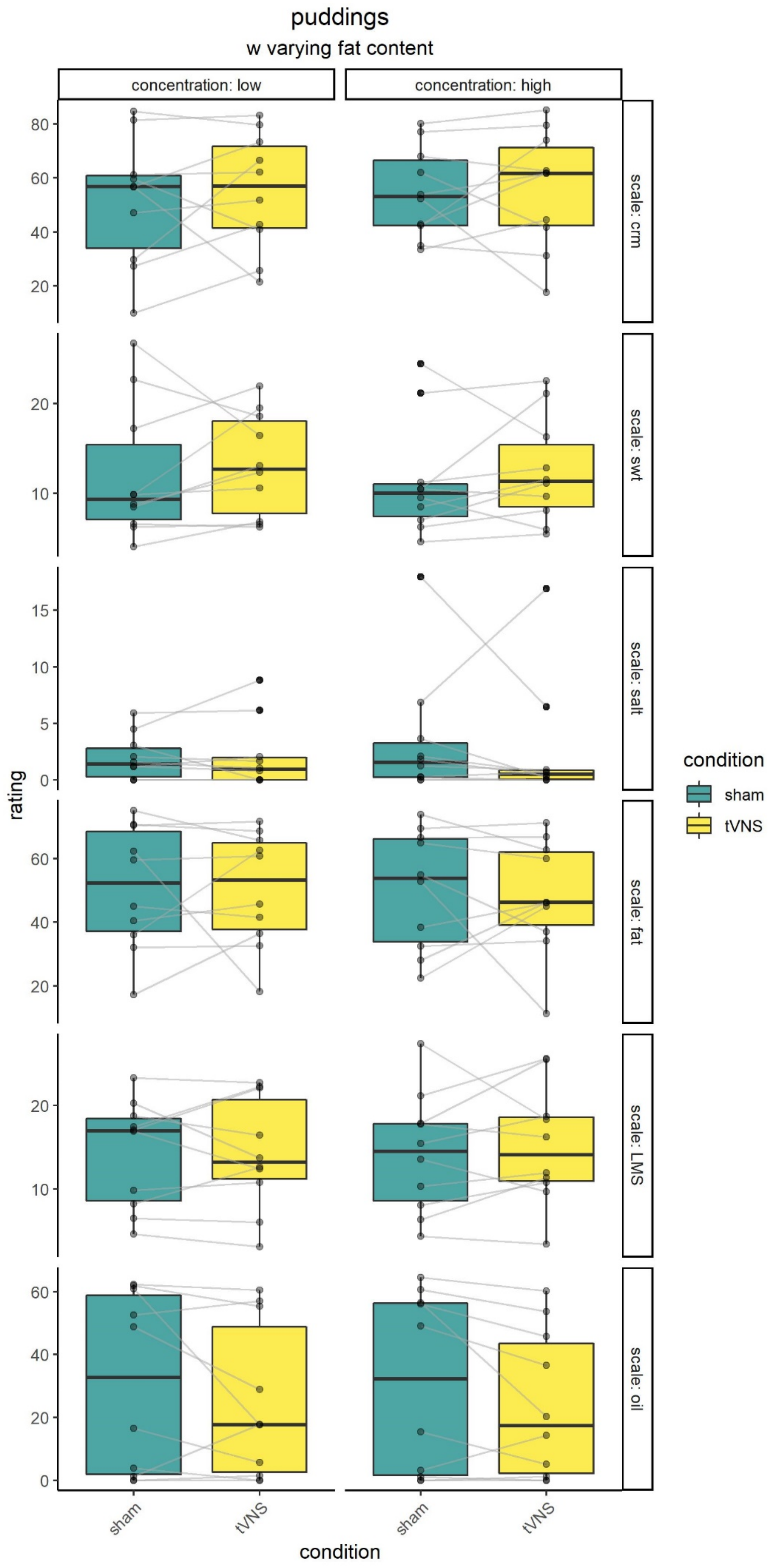
puddings
w varying fat content
concentration: low
concentration: high
scale: crm
scale: swt
scale: salt
scale: fat
scale: LMS
scale: oil
rating
condition
sham
tVNS
condition
sham
tVNS

Supplementary Figure 2: Ratings of puddings other than liking and wanting, included to control for "dumping": creaminess (crm), sweetness (swt), saltiness (salt), fattiness (fat), overall intensity (LMS) and oiliness (oil). Ratings under sham (dark green) vs tVNS (light green), plotted for the low fat puddings (left panel) and high fat puddings (right panel) separately. The boxplots elements as in Supplementary Figure 1.

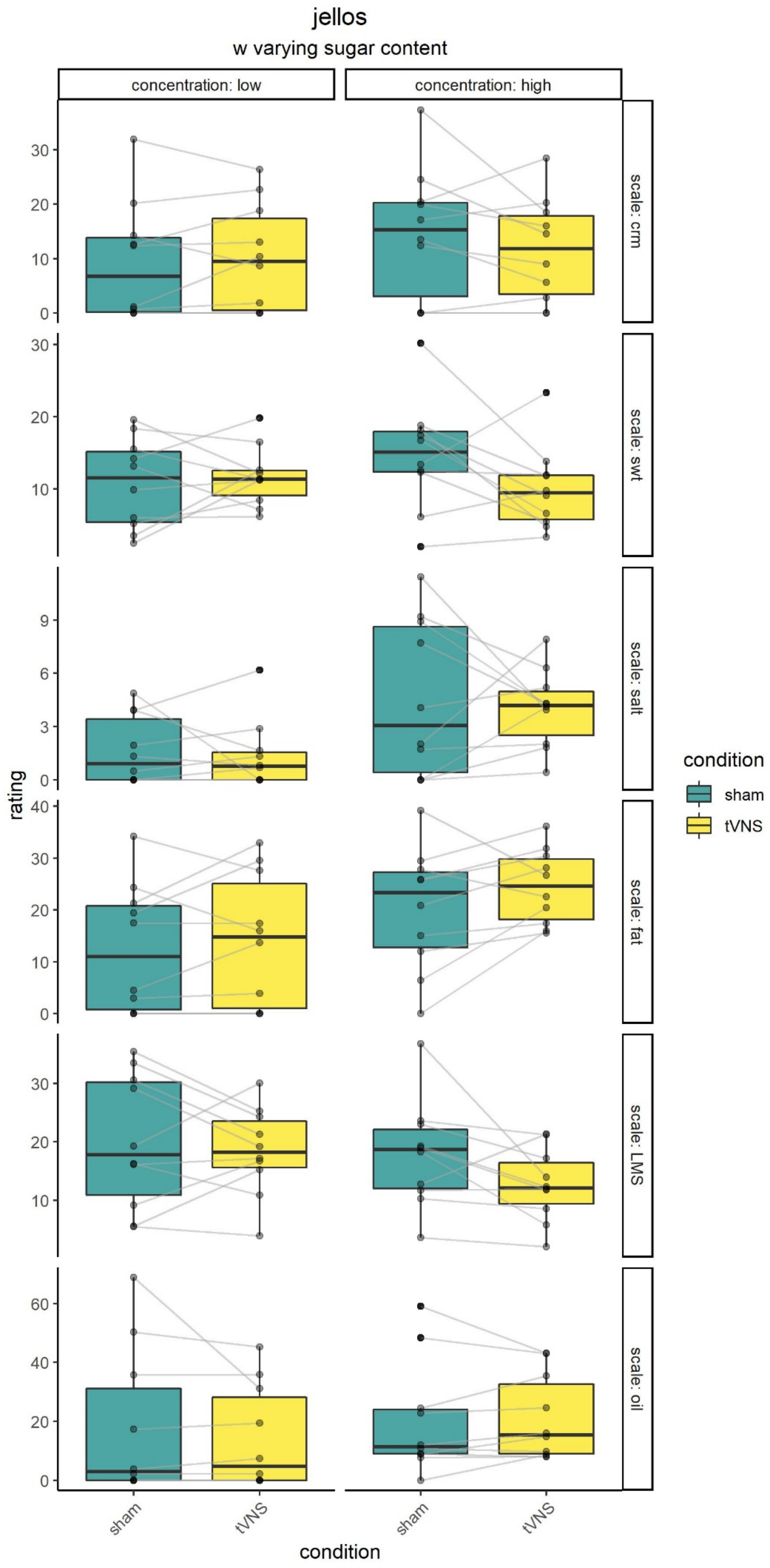
jellos
w varying sugar content
concentration: low
concentration: high
scale: crm
scale: swt
scale: salt
scale: fat
scale: LMS
scale: oil
rating
condition
sham
tVNS
condition
sham
tVNS

Supplementary Figure 3: Ratings of Jell-Os other than liking and wanting, included to control for "dumping": creaminess (crm), sweetness (swt), saltiness (salt), fattiness (fat), overall intensity (LMS) and oiliness (oil). Ratings under sham (dark green) vs tVNS (light green), plotted for the low sugar Jell-Os (left panel) and high sugar Jell-Os (right panel) separately. The boxplots elements as in Supplementary Figure 1.
